# Lead exposure and early child neurodevelopment among children 12–24 months in Kinshasa, the Democratic Republic of Congo

**DOI:** 10.1007/s00787-016-0860-3

**Published:** 2016-05-10

**Authors:** Espérance Kashala-Abotnes, Pépé Penghele Mumbere, Jeannette Mukanya Mishika, Ally Omba Ndjukendi, Davin Beya Mpaka, Makila-Mabe Guy Bumoko, Tharcisse Kalula Kayembe, Désiré Tshala-Katumbay, Théodore Kayembe Kazadi, Daniel Luwa E-Andjafono Okitundu

**Affiliations:** 1 Department of Neurology, Centre for Neuro-Psycho-Pathology, University of Kinshasa, Kinshasa, Democratic Republic of Congo; 2 Centre for International Health, Department of Global Public Health and Primary Care, University of Bergen, Bergen, Norway; 3 Department of Psychiatry, Centre for Neuro-Psycho-Pathology, University of Kinshasa, Kinshasa, Democratic Republic of Congo; 4 Centre for Research on Occupational and Environmental Toxicology, Oregon Health and Science University, Portland, OR USA

**Keywords:** Childhood, Lead exposure, Neurodevelopment, Temperament, Kinshasa/DRC

## Abstract

Childhood lead exposure remains a problem in developing countries, and little is known about its effects on early child neurodevelopment and temperament in the Democratic Republic of Congo (DRC). We, therefore, conducted this study to determine the association between lead exposure and the neurodevelopment and behaviour of children aged 12–24 months in Kinshasa, DRC. A cross-sectional study was conducted between February and June 2012, and parents of 104 children were invited to participate. Blood lead levels (BLLs) of each child were tested using the flame atomic spectrophotometry method. All children were subject to a clinical examination and assessed with two selected early child neurodevelopmental tools, the Gensini–Gavito and the baby characteristics questionnaire, to measure their neurodevelopment and temperament. Detectable BLLs ranged from 1 to 30 μg/dl with a geometric mean of 6.9 (SD 4.8) μg/dl. BLLs at 5–9 and ≥10 μg/dl were significantly associated with the child temperament (*p* <0.05). Perinatal and maternal factors did not seem to affect early child neurodevelopment and temperament. Children exposed to lead were reported with more temperament difficulties at even blood lead levels <10 μg/dl, suggesting the need for preventive and intervention measures to reduce lead exposure among children in Kinshasa, DRC.

## Background

Lead exposure remains an important public health challenge in the world, particularly in Africa due to, among other factors, poverty, low level of awareness and poor capacity to enforce legislation [1]. Lead exposure in early childhood have been associated with cognitive deficits, poor school performance and behavioural problems [2].

An increasing number of studies suggested that childhood lead exposure increases the risk for neurodevelopmental delays, behavioural problems and cognitive impairments even at blood lead levels below 10 μg/dl [3–5]. These impairments may persist into adolescence and adulthood, altering the optimal development of exposed children [6, 7].

Recently, the centers for disease control (CDC) eliminated the terminology “level of concern” and advised that children with blood lead levels requiring public health action should be identified using the reference value of 5 µg/dl. This value, generated by the national health and nutrition examination survey (NHANES), is based on the 97.5th percentile of blood lead level distribution among children aged 1–5 years old living in the United States of America (http://www.cdc.gov/mmwr/preview/mmwrhtml/mm6120a6.htm).

In the Democratic Republic of Congo (DRC), little is known about the effects of lead exposure on the neurodevelopment and behaviour of children in Kinshasa, and so far no studies have investigated such relation in the country. Findings from a recent study conducted in Kinshasa, suggested that a large proportion of children under 6 years of age were exposed to lead with a median blood lead levels of 11.5 μg/dl [8]. The authors reported that the use of leaded gasoline and activities of car battery recycling, such as handmade cooking utensils, appear to constitute the main source of lead exposure in the city of Kinshasa. The traditional use of fired clay for the treatment of gastritis by pregnant women was another significant contributor for elevated blood lead levels among children [8]. Although they highlighted the significant success achieved by the public health system with the removal of lead from gasoline since 2009, they emphasised the need for further research on the impact of childhood lead exposure [6, 9].

To the best of our knowledge, no studies have investigated the impact of childhood lead exposure in Kinshasa, and there is no established level for intervention with regard to lead exposure in DRC. There is, therefore, a need to study the adverse effects of lead exposure on early child neurodevelopment and behaviour in Kinshasa, DRC and to bring awareness among the population as a preventive measure. Our main objective was, therefore, to determine the association between lead exposure and neurodevelopment and behaviour of children aged 12–24 months living in Kinshasa, DRC. The specific objectives were to explore temperament and neurodevelopment of lead exposed children, and contributing factors such maternal psycho-affectivity and child early cerebral morbidity.

## Methods

### Participants and design

A cross-sectional study was conducted at one referral mother and child health care center in Kinshasa, DRC between February and June 2012. The center offers mother and child care and is located in one of the most vulnerable township of Kinshasa. Using convenience sampling, the parents/caregivers of children aged 12–24 months attending mother and child health care center were invited to participate in the study. Signed informed consent was obtained from parents/caregivers for each child.

#### Exclusion criteria

All children were examined by a physician. Those with medical history and/or clinical diagnosis of neurological condition such cerebral palsy or epilepsy, as well as children identified with clinical signs of undernutrition (marasmus and kwashiorkor) during the clinical examination and anthropometric measurements were excluded from the study.

### Measures and data collection

A structured interview was conducted with parents/caregivers to identify socio-demographic characteristics, usage of cooking utensils made with recycled lead based material, child birth and health history, maternal psycho-affectivity, factors of cerebral early morbidity, and neurobehavioural development of the child. Parental level of education was used as a proxy for socioeconomic status in this study. This was justified by the difficulty of assessing parental income, which may not objectively reflect the real socioeconomic situation in this township of Kinshasa.

#### Questionnaires

The Gensini–Gavito scale is a neurodevelopmental scale used to evaluate the psychomotor development of children in three domains, which are motor functions, communication/language and social adaptation [10]. The scale generates a psychomotor developmental quotient (PDQ) expressed in percentage. The PDQ is the ratio between the child real age (RA) obtained from his acquisition in all the three domains at the time of the examination and his chronological age (CA) times 100: PDQ = (RA/CA × 100). The scale is widely used in paediatric clinic in Kinshasa and has been locally validated at Kinshasa University Hospital [11]. The PDQ reported was used as continuous variable in the analysis.

The baby characteristics questionnaire (BCQ) is a brief 7-items questionnaire, based on the infant characteristics questionnaire (ICQ) developed by Bates et al. in 1979 [12]. The questionnaire invites parents/caregivers to indicate how they perceived the child temperament, focusing on difficult temperament, prone to irritability, agitation and negative response to restraints for each question on a scale from 1 (easy) to 7 (difficult). A total score is calculated by adding the scores of each question. A score below 35 indicates a perception as a child with an easy temperament, while a score ≥35 denotes a perception as a child with a difficult temperament. The scores reported were analysed as continuous variable in the analysis to compare children temperament between BLLs group’s categories.

#### Blood lead levels

Blood lead levels (BLLs) were tested using the flame atomic spectrophotometry method at the Research Centre for Geology and Mining in Kinshasa/DRC. The Centre participates in quality insurance programs from the British Geological Survey and the Korea Institute of Geosciences and Minerals Resources. The Venous blood was collected by venipuncture in an EDTA sealed tube and kept at low temperature (*T* 4 °C) and send for analysis at the Research Centre for Geology and Mining in Kinshasa/DRC. Samples were carefully handled to avoid contamination during the process of collection, transport and analysis. Control of contamination is done in relation to the lead standard solution (étalon) and the lab working environment, which does not allow external contamination by lead. Samples are treated in closed bombshell with inorganic acids in secured and controlled lab environment. BLLs were measured using the flame atomic absorption spectrophotometry with a detection range of 0.1 µg/dl for 5 µg of aqueous solution by the technique of extraction and prior concentration of the sample. The reference value of BLLs ≥5 µg/dl was used in this study as the country does not yet have a reference value for intervention in children. This value is chosen based on the value recommended by the advisory committee on childhood lead poisoning prevention (ACCLPP) of the centers for disease control and prevention (CDC) for children aged 1–5 years of age.

#### Usage of lead-containing cooking utensils 

During the structured interview with parents, they were asked whether or not they were using cooking utensils made from recycled car batteries for cooking the child’s food. These pots are commonly used in the area and have been identified as one of the common source of lead exposure in Kinshasa [6].

#### Clinical examination

Dr. Mumbere saw all children and performed their clinical examination. He was blinded to their BLLs.

#### Ethical considerations

Ethical approval was granted by the Institutional Review Board Ethics Commission for Human Research at the University of Kinshasa, DRC. Only children whose parents gave informed consent were invited to participate in the study.

### Statistical analysis

The statistical package for social sciences (SPSS) version 23.0 was used for data analysis. Descriptive analyses were used for sample characteristics. Since BLLs were not normally distributed, non-parametric analyses were used. Mean ranks were compared using Kruskal–Wallis test between groups. Kendall’s tau was used as measure of correlations, and generalised linear models (GLM) analyses with Poisson identity link were used to adjust for covariates, and to measure associations between risk factors at different BLLs. All tests were 2-tailed, and results were considered statistically significant when *p* values were less than or equal to 0.05.

## Results

### Subjects

Of the 104 children selected to participate in the study, six were excluded due to signs of malnutrition identified during the clinical examination. Four did not have detectable blood lead levels and nine mothers decline to participate. Biological parents, mainly mothers gave their informed consent and were the ones interviewed.

Totally 89 children with measurable BLLs were retained and included in the study.

### Socio-demographic characteristics

The children’s age varied between 12 and 24 months with a mean of 17.5 (SD 4.3). The family size ranged from 1 to 6 children per family with a mean of 2.8 (SD 1.7). There were more boys 52 (58.4 %) than girls 37 (41.6 %), but no gender differences were observed. More than half 48 (54 %) of the children were 18 months old and above. The majority of children 72 (81.2 %) had four siblings or less, and a total of sixty-one children (68.5 %) were the youngest sibling. Seventy-seven (86.5 %) fathers had a secondary and above level of education, while fewer mothers 66 (74 %) had a secondary and above level of education.

Regarding the context of lead exposure, 92 % reported to have lived in the area for more than 2 years, and 79 % reported using handmade cooking utensils made from recycled battery materials for cooking family and baby food.

### Maternal socioemotional characteristics and child medical history

The mean age of the parents at birth was 29.4 years (SD 6.5) for the mothers and 36.9 years (SD 6.3) for the fathers. There were also a good proportion of mothers reporting psycho-affective symptoms: undesired pregnancies (68 %) and lack of happiness in the early stage of the pregnancy (63.5 %). Almost all children (91 %) were born at term with a mean birthweight of 3291.5 grams (SD 593) and normal delivery. Only 25 % were exclusively breastfed up to 6 months. None were reported with risk factors for early cerebral morbidity. We did not find a statistically significant correlation between mother socioemotional characteristics (age at birth, pregnancy, labour, delivery, birthweight) and the child temperament and neurodevelopment as reported with the BCQ and Gensini–Gavito scale (Kendall’s correlation *p* value >0.05).

### Child early neurodevelopment and temperament

According to the mothers report, the PDQ mean percentage on the Gensini–Gavito scale was 90.68 (SD 14.0) and the mean score of the child temperament on the BCQ was 34.73 (SD 11.7). In general, 52 % of the children were reported with a neurodevelopment corresponding to their age according to the PDQ. Looking into the subscales of the PDQ, only 8 % of the children were reported as delayed on the motor neurodevelopment and 21 % on the communication/language development. More than half (58 %) were reported as delayed on the domain of social adaptation, suggesting social adaptation as the neurodevelopment domain where parents seem to report more difficulties.

On the evaluation of the child temperament as measured with the BCQ using a cut-offs score ≥35, a total of 59 children out of 89 (66 %) were reported and perceived as having difficult temperament by parents. This result might be in line with the delays in social adaptation as reported by parents.

### Blood lead levels

Blood lead levels (BLLs) ranged from 1 to 30 μg/dl with a geometric mean of 6.9 (SD 4.8) μg/dl. There was no significant mean difference between boys and girls 7.1 (SD 4.6) vs. 6.4 (SD 5.0), respectively (*p* >0.05).

Using three group categories of BLLs, proportions of children with BLLs between 0 and 4 μg/dl, 5–9 and ≥10μg/dl were, respectively 30 (34 %), 36 (40 %) and 23 (26 %). No gender differences were observed between the three group categories (*p* value >0.05).

### Blood lead levels and early child neurodevelopment

Table 1 summarises the findings of the child neurodevelopment as reported by parents on the Gensini–Gavito scale for BLLs analyses at 0–4, 5–9 and ≥10 μg/dl.

**Table 1:** Neurodevelopment and behaviour of 12–24 months old children as reported by parents in relation to blood lead levels. Results from a cross-sectional study on lead exposure among 89 children attending a mother and child care center in a vulnerable township of Kinshasa, Democratic Republic of Congo

	Blood lead levels μg/l		
	0–4	5–9	≥10
	*N* = 30	*N* = 36	*N* = 23
	Mean rank	Mean rank	Mean rank
**Neurodevelopment (gensini–gavito** **)**			
Psychomotor development quotient	44.98	47.08	41.76
Motor functions	45.90	43.72	45.83
Language and communication	44.18	44.44	46.93
Social adaptation	45.80	40.00	51.78
**Temperament (baby characteristics questionnaire)**			
Total difficult score (difficult temperament)	38.00	44.07	55.59*
Easily irritable	34.80	46.20	56.50*
Agitation, cries and disruptive temperament	35.60	45.70	56.20*
Rigorously cries and agitated when upset	37.10	45.50	54.50*
Plays nicely alone	42.50	45.00	48.22

* *p* value <0.05 (Kruskal–Wallis test*)*

As shown in the Table 1, no statistical significant differences were observed on the child neurodevelopment between the group categories of BLLs (Kruskal–Wallis *p* value >0.05).

Although no statistical significant difference was observed on the three domains of neurodevelopment of the child (motor, language and adaptation), children with BLLs at 5–9 µg/dl were reported with higher mean rank on the social adaptation compared to those with BLLs at 0–4 µg/dl (Kruskal–Wallis *p* value 0.053).

### Blood lead levels and child temperament

BLLs were significantly correlated with the child temperament (Kendall’s tau coef. 0.17; *p* value 0.02). Results from the BCQ on the child temperament as perceived by parents, showed significant differences between the group categories of BLLs at 0–4, 5–9 and ≥10 μg/dl. Indeed, more children with BLLs ≥5 μg/dl were perceived as difficult according to parents compared to those with BLLs between 0 and 4 µg/dl (Pearson Chi square *p* value 0.002). There was a significant higher proportion of children reported with difficult temperament among those with BLLs ≥10 μg/dl (22/23), and at 5–9 μg/dl (22/36), compared to those with BLLs at 0–4 μg/dl (15/30) (Pearson Chi square *p* value 0.002).

In addition, children with BLLs at 5–9 and ≥10 µg/dl were reported with higher mean rank on the BCQ (Kruskal–Wallis *p* value <0.05) (Table 1).

### Independent effects of blood lead levels on child neurodevelopment and temperament

We looked at the BLLs as a continuous variable, and found a significant correlation with the child temperament (Kendall’s tau coef. 0.17; *p* value 0.02). Similar correlation was observed when both the BLLs and the temperament were analysed as categorical variables for BLLs at, respectively 0–4, 5–9 and ≥10 μg/dl, and for the difficult temperament at cut-offs ≥35 (Kendall’s tau *p* value 0.001).

The independent effects of BLLs on the temperament of the child were analysed using the generalised linear models (GLM) Poisson link Identity analysis to adjust for potential confounders. The factors entered in the model and adjusted for, were risk factors related to the socioeconomic status, the mother and child medical history around pregnancy and birth, and the environmental exposure. This included the following variables: Mother and child antenatal, perinatal and postnatal risk factors (mother affectivity during pregnancy, duration of labour, circumstances of delivery, birthweight, parental age at child birth, morbidity, exclusive breastfeeding etc.), risk factors related to lead exposure and sociodemographic factors (number of years living in the area, daily usage of lead based utensils, family size, rank of the child).

After GLM analyses, a significant association was still observed between BLLs and the temperament of the child (*p* value 0.001), for both analyses as continuous and categorical variables (BCQ ≥35 and BLLs at 0–4, 5–9 and ≥10 µg/dl (coef. 4.3, *p* value 0.001).

## Discussion

The aim of this study was to determine the association between childhood lead exposure and the early child neurodevelopment and temperament among 12–24 month old children living in lead exposed environments in Kinshasa, DRC.

The mean BLLs was 6.9 μg/dl, which is considered high and above the recommended level of intervention. Results from this study suggest an association between childhood lead exposure and the child temperament among exposed children in Kinshasa, and support the need for comprehensive neurodevelopmental and behavioural assessments [8]. In addition, our results support and highlight the need for health prevention measures at primary care level among exposed children, as highlighted in previous studies from Kinshasa [9, 13].

To summarize, exposed children with BLLs ≥5 µg/dl significantly scored higher on the BCQ, meaning perceived as child with more difficult temperament. No significant association was found between BLLs and the child neurodevelopment. However, though neurodevelopment was not associated with BLLs, parents of children with BLLs ≥5 µg/dl reported more adaptation delays than those of children with BLLs <5 µg/dl. Surprisingly, mother psycho-affectivity during pregnancy did not seem to affect the child neurodevelopment or temperament.

In line with previous studies that have found associations between BLLs and the temperament of the child [4, 14–16], we found that exposed children with higher BLLs were reported to be more prone to irritability, agitation and negative response to restraints. Indeed, parents of children with BLLs at 5–9 µg/dl and ≥10 µg/dl reported to have greater difficulties in dealing with the child temperament compared to those at 0–4 μg/dl. We found temperamental difficulties at BLLs as early as 5–9 μg/dl as reported in previous studies [17, 18], and similar to studies reporting adverse events even at level <10 μg/dl [5, 19]. Based on the presence of adverse effects of childhood lead exposure on child temperament, urgent prevention and intervention measures are needed to reduce lead exposure among children in Kinshasa, DRC, as recommended by earlier studies [20].

Although parents of children with BLLs at 5–9 and at ≥10 µg/dl seem to report more delays in the domain of social adaptation, no significant neurodevelopmental difference was observed between children at different categories of BLLs. Our cross sectional design may have prevented us to find an association between level of lead exposure and the early child neurodevelopment. Indeed, child neurodevelopment is a continuous process that requires assessments at different stages of the child development. Longitudinal and follow-up studies would probably be more appropriate to detect early childhood neurodevelopmental delays at different developmental periods. However, delays reported in social adaptation may suggest neurodevelopmental issues that may occur and will be noticeable later in childhood. There is an urgent need to assess and comprehensively explore neurodevelopment of exposed children in Kinshasa, knowing that lead exposed children are at risk for neurodevelopmental delays and intellectual deficits later in their life [21].

Usage of lead-containing handmade kitchenware was the main reported source of exposure in about 80 % of the households. Although we did not explore other sources of exposure among children, the majority of mothers reported a daily usage of lead-containing kitchenware for cooking their baby food. It is well possible that in addition to these utensils, there are other contributing sources of contamination, previously described in Kinshasa, such as fired clay consumed during pregnancies, pesticides and paints [8]. Lead-containing kitchen utensils are commonly used as they are cheap, easily available, and they have been previously identified as one of the main source of lead exposure in Kinshasa. Because our results suggest adverse effects of lead exposure on the child temperament, longitudinal studies are needed to better explore the relation between daily lead exposure from handmade kitchen utensils and the child neurobehavioural development.

In contrast to other studies that have reported an association between affective characteristics of the mother and the child temperament [22, 23], we were unable to find such an association. Although the majority of mothers reported undesirable pregnancies with lack of happiness in its early stage, we did not use a screening tool to assess maternal anxiety and depressive symptoms during pregnancy, home environment or parenting style. Therefore we could not assume presence of depressive symptoms during pregnancy based on report of unhappiness feelings. It is, therefore, unsure whether or not mothers were depressed or have only reported a transient unhappiness, related to the non-desirability of the pregnancy in its early stage. In addition, only few mothers reported antenatal and perinatal risk factors (during pregnancies and deliveries), and no risk for early cerebral morbidity were identified among the studied children. This could explain why we were unable to find an association between maternal factors during pregnancy and delivery, child’s early cerebral morbidities and the child neurodevelopment and temperament. The lack of association between risk factors related to the mother, known as possible confounders, and the early child neurodevelopment and temperament, strengthens the consistency of our results. The association between BLLs and child difficult temperament, as perceived by parents, remained constant even after adjusted analysis and in absence of possible confounders.

This study has some methodological limitations associated with our study design and sample of convenience. Our results cannot be generalised to the whole population of Kinshasa or DRC, though they are of great importance and contribute to the knowledge and awareness of adverse effects of lead exposure on child behavioural development. The study, together with an earlier study conducted in Kinshasa, demonstrate how lead exposure, particularly among children, constitute an important concern for public health [6]. We used a cross-sectional design and tools that did not allow us to assess cognitive functioning of exposed children, which is an important aspect of child neurodevelopment. However, our primary objective was to explore whether there is an association between lead exposure and early child neurodevelopment and temperament. With the selected tools, the Gensini–Gavito scale and the BCQ, we were able to obtain useful informations on such an association. The assessments were, however, limited to the domains of motor functions, communication, language and social adaptation, and to the child temperament as perceived by parents. Comprehensive assessments of cognitive, intellectual functioning, school performance of exposed children in the later stage of their development and before their pre-school age are needed with different study designs (e.g., longitudinal studies). Another limitation is the limited number of covariates for which the analysis is controlled. We used parental education as a proxy for socioeconomic status (SES) as it is difficult to objectively measure the SES using parental income. This is due to the economic and political instability that have affected the DRC in the past decades, leaving the majority of the population still economically affected though the country is trying to reconstruct its economy.

The strength of the study resides in the awareness brought to better understand adverse effects of lead exposure on early child neurodevelopment and temperament of exposed children in Kinshasa, DRC. To the best of our knowledge, it is the first study investigating such an association among children in DRC. In addition, the study results will benefit policymakers in their formulation of integrated control policy against lead exposure for the protection of child health and their development to their potential of human capital. Dosage of BLLs should be considered among pregnant women consulting at antenatal care clinic and among children presenting with difficult temperament. Because handmade household kitchenware constitutes one of the major sources of lead exposure, it is important as preventive public health measures to inform the population on the associated risk. Preventive and intervention measures on lead exposure will contribute to the optimal wellbeing of exposed children in Kinshasa, DRC.

## Conclusions

Childhood lead exposure affects early child neurodevelopment and temperament of young children in Kinshasa, DRC. Results suggest the need for further investigations to explore the later in-life impact of childhood lead exposure on cognition, intellectual functioning, and school performance of pre- and school aged children.
